# Supplementary material for: Community perception towards mental illness and help-seeking intention in Southwest Ethiopian Peoples Regional State
Source: PLoS One. 2024 Oct 11;19(10):e0310512. doi: 10.1371/journal.pone.0310512 (PMC11469537; doi:10.1371/journal.pone.0310512)
Supplement: S1 Questionnaire — (DOCX) [file pone.0310512.s002.docx]

## English version Questionnaires

# Annexes

**Participants’ Information Sheet**

My name is………………We are collecting data on community perception towards mental illness for a research purpose. This form explains why we are going to do this study, your role in the study, the benefits and risks of involving in this study, compensations and confidentiality of the information you give us.

**Purpose-** This study is assessing Community Perception and Help Seeking intension towards Mental Illness And Its Associated Factors

**Procedures** - The researcher will conduct an interview and the interview will last about 20minutes. The interview will take place anywhere in the compound of the house, which is comfortable for the interview process and for the participant. You will be asked questions about socio-demographic issues, knowledge of mental health, exposure to mental illness or PWMI and attitude towards people with mental illness.

**Risks associated with the study-** Apart from the time you spend with us there we will not be any risk that you are exposed by participating in this study. If there is any question you don’t want to answer, you can miss out that question.

**Benefits of the study-** We expect that the study will help to withdraw attention of concerned bodies in order to emphasize more on community based strategies and interventions.

**Compensations-** There will not be any monetary incentive for your time.

**Confidentiality of your information-** The information you give during this study will be held confidential. Once the data is entered into a computer, it will be coded and becomes anonymous. Your personal information will never be disclosed in either oral or written form.

**Termination of the study-** You will only be selected based on your willingness and without any obligation to participate in the study. You have also the full right to withdraw your participation at any time before completing the interview.

I would also like to inform you that this study is approved by the ethical committees of mizan Tepi University college of health science.

## English version Questionnaires

**INSTRUCTION**: The questionnaire has 6 parts and it will take about 40-45 minutes to complete the interview. Thank you very much for your patience.

Kebeles code ___________ Questionnaire code ___________

Name of the data collector ________Date of interview __________Signature___________

Name of the supervisor ________ Date of supervision _________Signature___________

**Part I. Socio-demographic variables**

| No | Questions | answer |
| --- | --- | --- |
| 101 | What is your age? | ________________ |
| 102 | What is your gender? | 1. Male 2. Female |
| 103 | What is your religion? | 1. Orthodox 2. Muslim 3. Protestant |
| 104 | What is your Marital status? | 1. Single 2. Married 3. Divorced 4. Widowed/er 5. Separated |
| 105 | What is your educational status | 1. Unable to read& write 2. Abel to read & write only 3. Elementary school 4. Secondary school 5. Diploma & above |
| 106 | What is your occupation**?** | 1. Governmental 2. Private 3. Farmer 4. Housewife 5. Daily labourer |
| 107 | Where is your place of residence | 1. Urban 2. Rural |
| 108 | Household assets? | ----------------------- |

**Part II. Exposure to mental illness and history of mental illness information**

| S.no | Questions | Yes | No |
| --- | --- | --- | --- |
| 201 | Have you ever seen a person being scared by a people with mental illness? |  |  |
| 202 | Have you ever been scared by a person with mental illness? |  |  |
| 203 | Have you ever heard about mental illness on radio within the last year? |  |  |
| 204 | Have you ever heard about mental illness in religious places within the last year? |  |  |
| 205 | Have you ever seen/heard information about mental illness on TV within the last year? |  |  |
| 206 | Have you ever had family/relative with mental illness? |  |  |
| 207 | Have you ever had mental illness? |  |  |

**Part-III: Questions to assess mental health knowledge**

| No. | Questions | Strongly disagree | Disagree | Neutral | Agree | Strongly agree |
| --- | --- | --- | --- | --- | --- | --- |
| 301 | Most people with mental health problem want to have paid employment. |  |  |  |  |  |
| 302 | If a friend had a mental health problem, I know what advice to give them to get professional help. |  |  |  |  |  |
| 303 | Medication can be an effective treatment for people with mental health problem. |  |  |  |  |  |
| 304 | Psychotherapy (e.g. talk therapy or counselling) can be an effective treatment for people with mental health problem. |  |  |  |  |  |
| 305 | People with severe mental health problem can fully recover. |  |  |  |  |  |
| 306 | Most people with mental health problems go to a healthcare professionals to get help. |  |  |  |  |  |
| Say whether you think each condition is a type of mental illness by ticking one box only | | | | | | |
| 307 | Depression |  |  |  |  |  |
| 308 | Stress |  |  |  |  |  |
| 309 | Schizophrenia |  |  |  |  |  |
| 310 | Bipolar disorder( manic-depression) |  |  |  |  |  |
| 311 | Drug addiction |  |  |  |  |  |
| 312 | Grief |  |  |  |  |  |

**Part IV Community’s perception toward mental illness**

**(**Put “tick” sign under one of the five scales for each questions)

| S.No | Items | Strongly disagree | Disagree | Neutral | Agree | Strongly agree |
| --- | --- | --- | --- | --- | --- | --- |
| 301 | Do you agree that substance misuse like alcohol or drug could result in mental illness? |  |  |  |  |  |
| 302 | Do you agree that genetic inheritance could be the cause of mental illness? |  |  |  |  |  |
| 303 | Do you agree that head injury can be the cause of mental illness? |  |  |  |  |  |
| 304 | Do you agree that physical illness (like diabetes, HIV/AIDS) can be the cause of mental illness? |  |  |  |  |  |
| 305 | Do you agree that Schizophrenia is treatable? |  |  |  |  |  |
| 306 | Do you agree that stress in daily life can cause mental illness? |  |  |  |  |  |
| 307 | Do you agree that mental illness is contagious? |  |  |  |  |  |
| 308 | Do you agree that mental illness is punishment from God? |  |  |  |  |  |
| 309 | Do you agree that evil sprite can be the cause of mental illness? |  |  |  |  |  |

**Part-V questions to assess perceived causes of mental illness**

| Sr. no | Perceived causes of mental illness | yes | no |
| --- | --- | --- | --- |
| 401 | Stress |  |  |
| 402 | Poverty |  |  |
| 403 | Rumination |  |  |
| 404 | God’s punishment |  |  |
| 405 | Evil spirit |  |  |
| 406 | Sinful acts |  |  |
| 407 | Drug addiction |  |  |
| 408 | Physical illness |  |  |
| 409 | Germs |  |  |

**Parts VII help seeking behavior towards mental illness**

1. If you were having a personal or emotional problem, how likely is it that you would seek help from the following people?

| S.NO |  | Extremely Unlikely | Very Unlikely | Unlikely | Neutral | Likely | Very Likely | Extremely Likely |
| --- | --- | --- | --- | --- | --- | --- | --- | --- |
| 601 | Intimate partner (girlfriend, boyfriend, husband, wife) |  |  |  |  |  |  |  |
| 602 | Friend (not related to you) |  |  |  |  |  |  |  |
| 603 | Parent |  |  |  |  |  |  |  |
| 604 | Other relative/family member |  |  |  |  |  |  |  |
| 605 | Mental health professional (e.g. psychologist, social worker, counselor) |  |  |  |  |  |  |  |
| 606 | Health extension worker |  |  |  |  |  |  |  |
| 607 | Doctor/GP |  |  |  |  |  |  |  |
| 608 | Minister or religious leader (e.g. kes, Shek, Pastor) |  |  |  |  |  |  |  |
| 609 | I would not seek help from anyone |  |  |  |  |  |  |  |

2. If you were experiencing suicidal thoughts, how likely is it that you would seek help from the following people?

| S.NO |  | Extremely Unlikely | Very Unlikely | Unlikely | Neutral | Likely | Very Likely | Extremely Likely |
| --- | --- | --- | --- | --- | --- | --- | --- | --- |
| 611 | Intimate partner (girlfriend, boyfriend, husband, wife) |  |  |  |  |  |  |  |
| 612 | Friend (not related to you) |  |  |  |  |  |  |  |
| 613 | Parent |  |  |  |  |  |  |  |
| 614 | Other relative/family member |  |  |  |  |  |  |  |
| 615 | Mental health professional (e.g. psychologist, social worker, counselor) |  |  |  |  |  |  |  |
| 616 | Health extension worker |  |  |  |  |  |  |  |
| 617 | Doctor/GP |  |  |  |  |  |  |  |
| 618 | Minister or religious leader (e.g. kes, Shek, Pastor) |  |  |  |  |  |  |  |
| 619 | I would not seek help from anyone |  |  |  |  |  |  |  |

**Questions to assess mental social support**

|  |  | Alternative response/coding |
| --- | --- | --- |
| 701 | How many people are you so close to that you can count on them if you have great personal problems? | 1. None 2. 1-2 3. 3-5 4. 5 and above |
| 702 | How much interest and concern do people show in what you do? | 1. Very little 2. Little 3. Uncertain 4. Some 5. A lot |
| 703 | How easy is it to get practical help from neighbors if you should need it? | 1. Very difficult 2. Difficult 3. Possible 4. Easy 5. Very easy |
