# Supplementary material for: Community perception towards mental illness and help-seeking intention in Southwest Ethiopian Peoples Regional State
Source: PLoS One. 2024 Oct 11;19(10):e0310512. doi: 10.1371/journal.pone.0310512 (PMC11469537; doi:10.1371/journal.pone.0310512)
Supplement: Attachment — Submitted filename: plosone comments for percep.docx [file pone.0310512.s003.docx]

PLOS ONE

Community perception and help-seeking behavior towards mental illness in Southwest Ethiopia

Comments

1. Title should be written in capital for each word
2. Key word indicates about variable, so why southwestern Ethiopia is come to as a key word?
3. In abstract there is no clear statement of design, method and recommendation there is some ambiguity.
4. In introduction part there is inconstant word fluency and it does not address issue going be to study conducted
5. There is no clear contextual study from general to specific (no local study – global)
6. There is no clear gap is indicated. what the previous study was reviled about these research
7. In the Methodology part researcher not show the design approach, qual or quan or mixed and sampling is not clear
8. In data collection why interview is needed for large population? since the study is for large population?

In general the title of research is talk about perception community and help seeking of MI, but the researcher is talk about factors relate to MI, there is mismatch between title and discussion . It need some modification
