# Supplementary material for: Community perception towards mental illness and help-seeking intention in Southwest Ethiopian Peoples Regional State
Source: PLoS One. 2024 Oct 11;19(10):e0310512. doi: 10.1371/journal.pone.0310512 (PMC11469537; doi:10.1371/journal.pone.0310512)
Supplement: Attachment — Submitted filename: Health Seeking Behaviour Reveiwer Comments.docx [file pone.0310512.s004.docx]

Decision on Community perception and help-seeking behavior towards mental illness in Southwest Ethiopia

Dawit Getachew, MPH

**Comments from Reviewer**

Dear Authors,

Thank you for submitting the manuscript to the PLOS ONE journal. The manuscript is well written, though modifications and revisions are needed. The professional language edition is warranted as the manuscript needs synthesis and revision.

General: There are great inconsistencies and grammar issues.

There is a great discrepancy between the study topic and the results reported. The results are about recurrences of mental illness and its associated factors. Nothing was reported about the poor health seeking behavior of MI patients. Authors are suggested to update the study title to make it representative of the study results.

Title: The title is not specific, the author tried to address the health seeking behavior, and associated factors of MI at the same time which are unrelated and could be independent study topics. I suggest modifying the title to focus on either Health seeking behavior with associated factors. The title of the study also goes beyond its expected scope. The study was conducted in a single center (Mizan Tepi). However, the author(s) generalized the results for all Southwest Ethiopia. I suggest modifying the study scope. The title seems to that the study was conducted in single centers in Southwest Ethiopia; however, it is not associated with the study area. Hence, change the study area to a specific area.

# Abstract

- Avoid abbreviations in the abstract.
- This study was aimed to assess community perception and help seeking Intention towards mental illness in Southwest Ethiopia.
- Background: The abstract's background should discuss the background of the problem in the country context and show the study gaps. I recommend the author(s) to update it.
- Make I lower case and rewrite as intention.
- **Avoid Bold. Instead Make** A community-based
- **Results:** The results of the abstract were not written in line with the study objectives. Most of the results have a very wide confidence interval. How can the authors justify the wider CL?
- Arrange the keywords in alphabetical order i.e **Keywords**: Mental illness, Perception, help-seeking intention, Southwest Ethiopia

# Introduction

Indicate the prevalence of Mental illness in Ethiopia by number, not by prevalence. . fore example Depression, schizophrenia, attention deficit hyperactivity disorder (ADHD).Remove full-stop and make sentence case i.e. For example,

As for the knowledge gap, you have mentioned the delivery and implementation of the mental health

service were not successful in this study area. Would you indicate the number of studies published and the summary of their findings? Plus merge your citations. Therefore majority of the community prefers to seek treatment either from religious or spiritual healers for MI [15-21] [22]. illness[32, 34, 35] [24, 26]. merge your citations.

The last sentence of the introduction should state the aim of the study. I suggest Adding the research question.

# Methods

Methods: the methods part of the abstract did not indicate the sample size, and sampling technique used, and how the results of regression analysis were interpreted (the CI, OR, P-value). The author(s) mentioned, “data was collected by data collectors”. Who were those data collectors? It needs to be specified.

Study area: Make sure the information is valid and correct. The number of people with mental illness should be mentioned.

Separate the study design and population. The inclusion and exclusion criteria for the participants should be indicated.

## Sample size determination

It is not possible to assume that mental illness is directly related to medication. So why did you assume the p-of poor community perception towards MI as? There is study conducted in Ghimbi town,Ethiopia

Before you apply FPCF, the estimated sample size should be indicated. Why didn’t you include the estimate as it bears benefit for inference?

Why didn’t you use a simple random or systematic sampling technique?

Please indicate the psychometric properties of the MI.

Why post-data collection translation of the scale was not performed?

In order to give the participant confidentiality to their response, the interview will be conducted in separated room with protected privacy.Better to put under ethical consideration. But for knowledge score the higher the score indicate lower knowledge, thus score below and equal to the mean categorized as good knowledge. Why?

Operational Definitions: Use appropriate punctuation.

Data analysis

Please cite the literature that supports the p-value for bivariate logistic regression.

Results

What was the response rate?

Rather than saying ‘men’ it is better to say ‘males’

The description of socio-demographic characteristics needs to be longer; would you elaborate more?

Clinical characteristics of the study participants

Give an interpretation of the findings rather than simply mentioning the results. For example. Nearly, more than and etc.

Factor associated with Help seeking intention towards MI

Is there any justification for no associated factors with poor perception and help seeking intention towards mental illness?

For a p-value of 0.00, please report as P< 0.001. All p-values should be reported in three digits.

# Discussion

The discussion should start with the aim of the study and then mention the significant findings of the study. A total of 561 people with MI participated in this ambidirectional cross-sectional study. What does it mean?

Paragraph 1; It may be beneficial if you rewrite.

This discrepancy in the outcome can be the result of using a different data collection technique or sample size. Is there any evidence showing this conclusion?

Regarding the factors associated, you should only discuss those significantly associated.

It would be better if you cite to make strong arguments.

What are the implications of the study?

Limitations of the study

You have listed several limitations. Do you think these limitations affect the validity of your study? I think there are strengths of the study; hence, need to add.

Conclusion

Did you calculate the proportion or the score?

Is there any recommendation?

# References

Revise references. Follow the Vancouver citation style.
