# Supplementary material for: Community perception towards mental illness and help-seeking intention in Southwest Ethiopian Peoples Regional State
Source: PLoS One. 2024 Oct 11;19(10):e0310512. doi: 10.1371/journal.pone.0310512 (PMC11469537; doi:10.1371/journal.pone.0310512)
Supplement: Attachment — Submitted filename: Comment for PLoS ONE.docx [file pone.0310512.s005.docx]

**PONE-D-23-13743
Community perception and help-seeking behavior towards mental illness in Southwest Ethiopia.**

First I would like to say thank you for the opportunity to review the paper. The title is so interesting and I have points that need clarifications and improvements.

**General comments**

1. Unnecessary capitalizations. E.g.: line 25, and others and do proofreading accordingly.
2. Background: editorial comments like unnecessary full stops, and inappropriate citations.
3. The data collection tool needs citation under the method: data collection tool section.
A. Under the background, your study gap is not enough to conduct the study and needs improvement.
B. Sample size: Adding non-response or multiplying by design effect…which comes first? And again your sample size seems incorrect. I need clarification.
C. Sample size: clearly show the necessary steps for both the first and second objectives.
D. What is your reference regarding variable categorization, for example under socio-demographic characteristics like age?
E. Table 3: why it is necessary to put variables like socio-demographic characteristics in the table?
F. Strength and limitation:

- Study design cannot be a limitation of your study.
- The title by itself cannot be the strength of the study.

G. The result in the figure and the document is different and it seems fabricated.
