# Supplementary material for: Community perception towards mental illness and help-seeking intention in Southwest Ethiopian Peoples Regional State
Source: PLoS One. 2024 Oct 11;19(10):e0310512. doi: 10.1371/journal.pone.0310512 (PMC11469537; doi:10.1371/journal.pone.0310512)
Supplement: Attachment — Submitted filename: Response to Reviewers.docx [file pone.0310512.s006.docx]

**Point by point response to the reviewer comment**

**Editor comment**

PONE-D-23-13743

Community perception and help-seeking behavior towards mental illness in Southwest Ethiopia

PLOS ONE

Dear Dr. Getachew ,

Thank you for submitting your manuscript to PLOS ONE. After careful consideration, we feel that it has merit but does not fully meet PLOS ONE’s publication criteria as it currently stands. Therefore, we invite you to submit a revised version of the manuscript that addresses the points raised during the review process.

Please submit your revised manuscript by Jul 24 2024 11:59PM. If you will need more time than this to complete your revisions, please reply to this message or contact the journal office at <a href="mailto:plosone@plos.org">plosone@plos.org. When you're ready to submit your revision, log on to https://www.editorialmanager.com/pone/ and select the 'Submissions Needing Revision' folder to locate your manuscript file.

Please include the following items when submitting your revised manuscript:

- A rebuttal letter that responds to each point raised by the academic editor and reviewer(s). You should upload this letter as a separate file labeled 'Response to Reviewers'.
- A marked-up copy of your manuscript that highlights changes made to the original version. You should upload this as a separate file labeled 'Revised Manuscript with Track Changes'.
- An unmarked version of your revised paper without tracked changes. You should upload this as a separate file labeled 'Manuscript'.

**Author response:** thank you dear editor, we extremely appreciate your support and also all reviewers for their comments and question. In the revised manuscript we have tried to address all the comments and points raised by the editors, reviewers and also the journal requirement.

If you would like to make changes to your financial disclosure, please include your updated statement in your cover letter.

**Author response:** thank you dear editor regarding the financial disclosure we are waivered by the request we made on the first submission and we are not going to change that.

Guidelines for resubmitting your figure files are available below the reviewer comments at the end of this letter.

**Author response:** thank you dear editor we have prepared and submitted the figure file as per PLOS guideline for figure file submission.

If applicable, we recommend that you deposit your laboratory protocols in protocols.io to enhance the reproducibility of your results. Protocols.io assigns your protocol its own identifier (DOI) so that it can be cited independently in the future. For instructions see: <https://journals.plos.org/plosone/s/submission-guidelines#loc-laboratory-protocols>. Additionally, PLOS ONE offers an option for publishing peer-reviewed Lab Protocol articles, which describe protocols hosted on protocols.io. Read more information on sharing protocols at <https://plos.org/protocols?utm_medium=editorial-email&utm_source=authorletters&utm_campaign=protocols>.
**Author response:** thankyou dear editors this is not applicable on the current manuscript.

We look forward to receiving your revised manuscript.

Kind regards,

Firomsa Bekele Negera, Msc
Academic Editor
PLOS ONE

**Journal requirements:**

When submitting your revision, we need you to address these additional requirements.

1. Please ensure that your manuscript meets PLOS ONE's style requirements, including those for file naming. The PLOS ONE style templates can be found at
https://journals.plos.org/plosone/s/file?id=wjVg/PLOSOne_formatting_sample_main_body.pdf and
https://journals.plos.org/plosone/s/file?id=ba62/PLOSOne_formatting_sample_title_authors_affiliations.pdf

**Author response**: Thank you dear editor we have prepared the revised manuscript based on the journal requirement using The PLOS ONE style templates.

2. PLOS ONE does not copy edit accepted manuscripts (https://journals.plos.org/plosone/s/criteria-for-publication#loc-5). To that effect, please ensure that your submission is free of typos and grammatical errors.

**Author response:** Thank you dear editor we have copy edited the manuscript to make it free of typos and grammatical error.

3. We note that the grant information you provided in the ‘Funding Information’ and ‘Financial Disclosure’ sections do not match.

**Author response:** Thank you dear reviewer we have accepted the comment and corrected on the revised manuscript.

When you resubmit, please ensure that you provide the correct grant numbers for the awards you received for your study in the ‘Funding Information’ section.

**Author response:** Thank you dear reviewer we have accepted the comment and corrected on the revised manuscript.

3. In the online submission form, you indicated that [IThe data set can be accessible from the corresponding author up on reasonable request.]. 
All PLOS journals now require all data underlying the findings described in their manuscript to be freely available to other researchers, either 1. In a public repository, 2. Within the manuscript itself, or 3. Uploaded as supplementary information.
This policy applies to all data except where public deposition would breach compliance with the protocol approved by your research ethics board. If your data cannot be made publicly available for ethical or legal reasons (e.g., public availability would compromise patient privacy), please explain your reasons on resubmission and your exemption request will be escalated for approval.

**Author response:** Thank you dear editor we have uploaded the data set as supplementary material

4. Your ethics statement should only appear in the Methods section of your manuscript. If your ethics statement is written in any section besides the Methods, please move it to the Methods section and delete it from any other section. Please ensure that your ethics statement is included in your manuscript, as the ethics statement entered into the online submission form will not be published alongside your manuscript.

**Author response:** thank you dear editor we have placed the ethics statement in methods and material section.

**Reviewer 1 comment.**

PLOS ONE

Community perception and help-seeking behavior towards mental illness in Southwest Ethiopia

Comments

**Author response:** Thank you dear reviewer, we have accepted the comment and corrected the title in the revised manuscript.

**‘Community Perception towards Mental Illness and Help Seeking Intention in Southwest Ethiopian Peoples Regional State’** (page number 1, line number 1-2).

1. Title should be written in capital for each word

**Author response:** Thank you dear reviewer, we have accepted the comment and corrected the title on the revised manuscript. **‘Community Perception towards Mental Illness and Help Seeking Intention in Southwest Ethiopian Peoples Regional State’** (Page number 1, Line number 1-2).

2. Key word indicates about variable, so why southwestern Ethiopia is come to as a key word?

**Author response:** Thank you dear reviewer, we have omitted southwest Ethiopia from the keyword and we have included pertinent variable as a keyword.

Keywords: Help seeking intention, Mental illness information, Perception of Mental illness. (Page number 2, Line number 47).

3. In abstract there is no clear statement of design, method and recommendation there is some ambiguity.

**Author response:** Thank you dear reviewer, we have rewritten the Abstract by clearly describing the design, methods and implication of the finding and recommendations.

*(Page number 2, Line number 36-46).*

4. In introduction part there is inconstant word fluency and it does not address issue going be to study conducted

**Author response:** Thankyou dear reviewer we have accepted your comment and we have copy edited the introduction section of the manuscript. Therefore, the current study can address the issue studied more clearly. *(Page number 3, Line number 49-71).*

5. There is no clear contextual study from general to specific (no local study – global)

**Author response:** Thankyou dear reviewer we have shown the context of the study from general to specific in the revised manuscript. *(Page number 3, Line number 49-71).*

6. There is no clear gap is indicated. what the previous study was reviled about these research

**Author response:** Thankyou dear reviewer we have included what previous research reviled about communities’ perception towards mental illness and help seeking behaviour for mental illness.

7. In the Methodology part researcher not show the design approach, qual or quan or mixed and sampling is not clear

**Author response:** Thank you dear reviewer we have accepted your comment in the methods and material section of revised manuscript we have clearly putted the design of this research was quantitative. *(Page number 4, Line number 80).*

Also, we have described the sampling technique was a multistage sampling technique and we have showed how study participant were selected by including a sampling distribution scheme. *(Page number 5, Line number 109-112).*

8. In data collection why interview is needed for large population? since the study is for large population?

**Author response:** Thank you dear reviewer we have used interview method because majority of the participant cannot read and write *(Page number 7, Line number 112).*

**Reviewer comment:** In general, the title of research is talk about perception community and help seeking of MI, but the researcher is talk about factors relate to MI, there is mismatch between title and discussion. It needs some modification

**Author response:** Thank you dear reviewer we have accepted your comment and we have edited the manuscript by focusing on our primary and secondary outcome perception community and help seeking of MI. *(Page number 3, Line number 49-71).*

**Reviewer 2 comment.**

Decision on Community perception and help-seeking behavior towards mental illness in Southwest Ethiopia

Dawit Getachew, MPH

**Comments from Reviewer**

Dear Authors,

Thank you for submitting the manuscript to the PLOS ONE journal. The manuscript is well written, though modifications and revisions are needed. The professional language edition is warranted as the manuscript needs synthesis and revision.

**Author response:** Thank you dear reviewer we accept the comment we have copy edited the manuscript to correct all grammatical and spelling error that obscured the easy communication and correct message of the paragraph.

**Comments from Reviewer:** General: There are great inconsistencies and grammar issues.

**Author response:** Thank you dear reviewer we have corrected all the inconsistencies and grammatical error in the revised manuscript.

**Comments from Reviewer:** There is a great discrepancy between the study topic and the results reported. The results are about recurrences of mental illness and its associated factors. Nothing was reported about the poor health seeking behavior of MI patients. Authors are suggested to update the study title to make it representative of the study results.

**Author response:** Thank you dear reviewer, we appreciate the comment and we wan to apologies for the inconvenience. However, this study assessed healthy individuals regarding their perception and their help seeking intention towards mental illness. But we have copy edited the whole manuscript to make clear the information conveyed.

**Comments from Reviewer:** Title: The title is not specific, the author tried to address the health seeking behavior, and associated factors of MI at the same time which are unrelated and could be independent study topics. I suggest modifying the title to focus on either Health seeking behavior with associated factors. The title of the study also goes beyond its expected scope. The study was conducted in a single center (Mizan Tepi). However, the author(s) generalized the results for all Southwest Ethiopia. I suggest modifying the study scope. The title seems to that the study was conducted in single centers in Southwest Ethiopia; however, it is not associated with the study area. Hence, change the study area to a specific area.

**Author response:** Thank you dear reviewer, we appreciate your genuine comment. Regarding the title of this study, it is about Community Perception towards Mental Illness and Help Seeking Intention among the general population. We agree with you the manuscript can be written as independent article. However, it’s also verry advantageous for planner, policy maker and even for researcher to find both information at one place.

Also, this study was a community based multi-centre which address four zone of Southwest Ethiopian people’s regional state including 11 district administration and four city administrations. The finding can represent all 57 administrative structure of the six zone of Southwest Ethiopian people’s regional state as we have used multistage sampling and also multiplied the sample size with design effect.

**Comments from Reviewer:**

# Abstract

- Avoid abbreviations in the abstract.

**Author response:** Thank you dear reviewer we have removed all the abbreviation from the abstract section of the manuscript.

**Comments from Reviewer:**

- This study was aimed to assess community perception and help seeking Intention towards mental illness in Southwest Ethiopia.

**Author response:** Yes, dear reviewer this study was aimed to assess community perception and help seeking Intention towards mental illness in Southwest Ethiopia.

**Comments from Reviewer:**

- Background: The abstract's background should discuss the background of the problem in the country context and show the study gaps. I recommend the author(s) to update it.

**Author response:** Thank you dear reviewer we have updated the back ground of the abstract section by discussing the problem under investigation.

**Comments from Reviewer:**

- Make I lower case and rewrite as intention.
- **Avoid Bold. Instead Make** A community-based

**Author response:** thank you dear reviewer we apologies for all the incorrect spelling we have correct in the revised manuscript. We have also omitted unnecessary Bold case.

**Comments from Reviewer:**

- **Results:** The results of the abstract were not written in line with the study objectives. Most of the results have a very wide confidence interval. How can the authors justify the wider CL?

**Author response:** Thank you dear reviewer, we have corrected the result subsection of the abstract. We have showed the magnitude of community perception and help seeking intention towards mental illness in the study area. Then we have shown the factors associated with community perception and help seeking intention. *(Page number 2, Line number 36-43).*

**Comments from Reviewer:**

- Arrange the keywords in alphabetical order i.e **Keywords**: Mental illness, Perception, help-seeking intention, Southwest Ethiopia

**Author response:** thank you dear reviewer for your comment we have arranged the keyword in alphabetical order, in addition we have updated the key word as per reviewer one comment

**Keywords:** Help seeking intention, Mental illness information, Perception of Mental illness. (Page number 2, Line number 47).

**Comments from Reviewer:**

# Introduction

Indicate the prevalence of Mental illness in Ethiopia by number, not by prevalence. . fore example Depression, schizophrenia, attention deficit hyperactivity disorder (ADHD).Remove full-stop and make sentence case i.e. For example,

As for the knowledge gap, you have mentioned the delivery and implementation of the mental health service were not successful in this study area. Would you indicate the number of studies published and the summary of their findings? Plus merge your citations. Therefore majority of the community prefers to seek treatment either from religious or spiritual healers for MI [15-21] [22]. illness[32, 34, 35] [24, 26]. merge your citations.

**Author response:** thank you dear reviewer **We have presented the prevalence of Mental illness disorders in Ethiopia.**

**Comments from Reviewer:**

The last sentence of the introduction should state the aim of the study. I suggest Adding the research question.

**Author response:** Thankyou dear reviewer we have accepted the comment and we have added the aim of the study on the last line of the introduction section**. ‘There for this study aimed to assess community perception towards MI and communities help seeking intention in SWEPRS**’ *(Page number 3, Line number 69-70).*

**Comments from Reviewer:**

# Methods

Methods: the methods part of the abstract did not indicate the sample size, and sampling technique used, and how the results of regression analysis were interpreted (the CI, OR, P-value). The author(s) mentioned, “data was collected by data collectors”. Who were those data collectors? It needs to be specified.

**Author response:** Thankyou dear reviewer we have included the sample size, and sampling technique used, and how the results of regression analysis were interpreted (the CI, OR, P-value) in the revised manuscript.

**Comments from Reviewer:**

Study area: Make sure the information is valid and correct. The number of people with mental illness should be mentioned.

**Author response:** thank you dear reviewer we have accepted the comment and we have made sure the information is correct, but we can not find the number of people with menta illness in the region.

**Comments from Reviewer:**

Separate the study design and population. The inclusion and exclusion criteria for the participants should be indicated.

**Author response:** Thank you dear reviewer we have accepted the comment and we have wrote the study design and population in different sub section of methods and material section of the manuscript. *(Page number 4, Line number 79-85).*

**Comments from Reviewer:**

## Sample size determination

It is not possible to assume that mental illness is directly related to medication. So why did you assume the p-of poor community perception towards MI as? There is study conducted in Ghimbi town,Ethiopia Before you apply FPCF, the estimated sample size should be indicated. Why didn’t you include the estimate as it bears benefit for inference?

**Author response:** Thank you dear reviewer, we really appreciate your concern. However, the previous study was conducted in Gimbi town of west wollega zone Oromia regional state, this study area is bordered with zone which have multiple referral hospital. But, this study was conducted in area where one referral hospital is located in the region and also the study included population from rural district, pastoral district and few urban areas. Therefore, its difficult to use the proportion of community perception from Gimbi town. Moreover, this study used the sample size calculated for the second objective.

**Comments from Reviewer:**

Why didn’t you use a simple random or systematic sampling technique?

**Author response:** dear reviewer thank you for question; we used multi stage sampling because we have to follow several stage to select participant from the whole region**.**

**Comments from Reviewer:**

Please indicate the psychometric properties of the MI.

**Author response: thank you dear reviewer if your question was about ‘**for psychometric properties of the mental health knowledge schedule question’ we have presented the result on page number 10, line number 192-110

**Comments from Reviewer:**

Why post-data collection translation of the scale was not performed?

In order to give the participant confidentiality to their response, the interview will be conducted in separated room with protected privacy. Better to put under ethical consideration. But for knowledge score the higher the score indicate lower knowledge, thus score below and equal to the mean categorized as good knowledge. Why?

**Author response:** Thank you dear reviewer we have accepted the comment and corrected it on the revised manuscript.

**Comments from Reviewer:**

Operational Definitions: Use appropriate punctuation.

**Author response:** Thank you dear reviewer we have accepted the comment and corrected on the revised manuscript.

**Comments from Reviewer:**

Data analysis

Please cite the literature that supports the p-value for bivariate logistic regression.

**Author response:** Thank you dear reviewer we have accepted the comment and we have cited literature that supports the p-value for bivariate logistic regression on the revised manuscript.

**Comments from Reviewer:**

Results:

What was the response rate?

**Author response:** Thank you dear reviewer, the response rate of this study was 95.4%. *(Page number 6, Line number 151).*

**Comments from Reviewer:**

Rather than saying ‘men’ it is better to say ‘males’

**Author response:** Thank you dear reviewer we have accepted the comment and corrected. *(Page number 6, Line number 152).*

**Comments from Reviewer:**

The description of socio-demographic characteristics needs to be longer; would you elaborate more?

**Author response:** Thank you dear reviewer we have accepted the comment and corrected on the revised manuscript.

**Comments from Reviewer:**

Clinical characteristics of the study participants

**Author response:** Thank you dear reviewer for your concern, however this manuscript was only about perception of were general population regarding mental illness.

**Comments from Reviewer:**

Give an interpretation of the findings rather than simply mentioning the results. For example. Nearly, more than and etc.

**Author response:** Thank you dear reviewer we have accepted the comment and corrected writing the result by adding interpretation in the revised manuscript.

**Comments from Reviewer:**

Factor associated with Help seeking intention towards MI

Is there any justification for no associated factors with poor perception and help seeking intention towards mental illness?

**Author response:** Thank you dear reviewer we have accepted the comment and corrected on the revised manuscript.

**Comments from Reviewer:**

For a p-value of 0.00, please report as P< 0.001. All p-values should be reported in three digits.

**Author response:** Thank you dear reviewer, to keep the size of the table on one page its more appropriate if we describe the P value using single and double Asterix.

**Comments from Reviewer:**

# Discussion

The discussion should start with the aim of the study and then mention the significant findings of the study. A total of 561 people with MI participated in this ambidirectional cross-sectional study. What does it mean?

**Author response:** your apology dear reviewer I am not clear with this question.

**Comments from Reviewer:**

Paragraph 1; It may be beneficial if you rewrite.

This discrepancy in the outcome can be the result of using a different data collection technique or sample size. Is there any evidence showing this conclusion?

**Author response:**

**Comments from Reviewer:**

Regarding the factors associated, you should only discuss those significantly associated.

It would be better if you cite to make strong arguments.

**Author response:** Thank you dear reviewer we have discussed only significantly associated variables and we have tried to make strong argument with finding from previous studies.

**Comments from Reviewer:**

What are the implications of the study?

**Author response:** The implication of this study is majority of adult in the study area lack good attitude and thy also have unfavourable help seeking intention due to lack of appropriate information and being from rural side of the country. This shows urgent action is needed to improve the perception and help seeking intention otherwise it can result stigma towards mentally ill peoples and also individual with mental disorder tend to seek care from non-professionals. This can result increasing prevalence of the disease and also exacerbate the suffering of individual with mental illness.

**Comments from Reviewer:**

Limitations of the study

You have listed several limitations. Do you think these limitations affect the validity of your study? I think there are strengths of the study; hence, need to add.

**Author response:** Thank you dear reviewer we have added the strength of the limitation. *(Page number 14 Line number 260-261).*

**Comments from Reviewer:**

Conclusion

**Author response:**

**Comments from Reviewer:**

Did you calculate the proportion or the score?

**Author response:**

**Comments from Reviewer:**

Is there any recommendation?

**Author response:** yes, dear reviewer we recommend **‘**To improve the community MI perception and help seeking intention providing appropriate and tailored information regarding the cause, type and severity of MI particularly focusing the rural and who have experienced MI from in their family, relative or friends. This can be achieved through integrating the community mental health education initiatives and community mental health services in to existing Ethiopia's primary health care system.**’** *(Page number 14 Line number 268-273).*

**Comments from Reviewer:**

# References

Revise references. Follow the Vancouver citation style.

**Author response:** Thank you dear reviewer we have updated the reference we have used Vancouver citation style. *(Page number 16-17, Line number 306-391).*

**Reviewer 3 comment.**

**PONE-D-23-13743
Community perception and help-seeking behavior towards mental illness in Southwest Ethiopia.**

First I would like to say thank you for the opportunity to review the paper. The title is so interesting and I have points that need clarifications and improvements.

**General comments**

1. Unnecessary capitalizations. E.g.: line 25, and others and do proofreading accordingly.

**Author response:** Thank you dear editor we have accepted the comment and corrected the grammatical and spelling error in the revised manuscript.

2. Background: editorial comments like unnecessary full stops, and inappropriate citations.

**Author response:** Thank you dear editor we have accepted the comment and corrected the punctuations and updated our citation. *(Page number 3, Line number 49-71).*

3. The data collection tool needs citation under the method: data collection tool section.

**Author response:** Thank you dear reviewer we have cited from where we get the data collection tool method: data collection tool section. *(Page number 5, Line number 118).*

A. Under the background, your study gap is not enough to conduct the study and needs improvement.

**Author response:** thankyou dear editor we have elaborated the gap justifying the conduct of this research in the revised manuscript. *(Page number 3, Line number 70-71).*

B. Sample size: Adding non-response or multiplying by design effect…which comes first? And again your sample size seems incorrect. I need clarification.

**Author response:** Dear editor hank you for your concern we have add the non-response after multiplying with the design effect. *(Page number 4-5, Line number 91-107).*

C. Sample size: clearly show the necessary steps for both the first and second objectives.

**Author response:** thank you dear reviewer for your comment we have clearly shown necessary steps for both objective on the revised manuscript. *(Page number 4-5, Line number 91-107).*

D. What is your reference regarding variable categorization, for example under socio-demographic characteristics like age?

**Author response:** Thank you dear reviewer we categorized sociodemographic variables following categorization used by similar studies so that we can easily compare the finding with previously done research.

For example, the age categorization was previously used by study done in Ethiopia Gimbi town.

E. Table 3: why it is necessary to put variables like socio-demographic characteristics in the table?

**Author response:** Thankyou dear reviewer we have accepted the comment and corrected on the revised manuscript.

F. Strength and limitation:

- Study design cannot be a limitation of your study.
- The title by itself cannot be the strength of the study.

**Author response:** Dear reviewer we really appreciate your comment we have corrected the limitation section of this study by addressing how the research tried to control the possible limitation. *(Page number 13, Line number 253-258).*

G. The result in the figure and the document is different and it seems fabricated.

**Author response:** Thank you dear reviewer we have accepted the comment and corrected on the revised manuscript.
